# The relationship between severe extracranial artery stenosis or occlusion and cerebral small vessel disease in patients with large artery atherosclerotic cerebral infarction

**DOI:** 10.3389/fneur.2022.1008319

**Published:** 2022-11-04

**Authors:** Pei Dai, Hui-xian Yu, Zhao-xia Wang, Si-hao Liu, Guang-qing Xu

**Affiliations:** ^1^Department of Rehabilitation Medicine, Beijing Tiantan Hospital, Capital Medical University, Beijing, China; ^2^China National Clinical Research Center for Neurological Diseases, Beijing, China; ^3^Department of Rehabilitation Medicine, Guangdong Provincial People's Hospital, Guangdong Academy of Medical Sciences, Guangzhou, China

**Keywords:** severe extracranial artery stenosis, occlusion, large atherosclerotic cerebral infarction, total cerebral small vessel disease scores, Fazekas scores

## Abstract

**Background and purpose:**

Extracranial artery stenosis (ECAS) is associated with the presence of individual markers of cerebral small vessel disease (CSVD). Here, we investigated the relationship between severe extracranial artery stenosis or occlusion and CSVD in patients with large artery atherosclerotic (LAA) cerebral infarction.

**Methods:**

A total of 128 patients with LAA cerebral infarction who met our specific inclusion criteria were selected, including 92 males and 36 females. These patients were divided into three groups based on whether they had severe symptomatic extracranial arterial stenosis or occlusion, severe asymptomatic extracranial artery stenosis or occlusion, or severe extracranial artery stenosis or occlusion (both symptomatic and asymptomatic). Intra-group comparisons were then performed to examine whether there were any differences in the total CSVD scores and Fazekas scores.

**Results:**

Patients with severe extracranial arterial stenosis or occlusion and those with severe asymptomatic extracranial arterial stenosis or occlusion had a significantly higher total CSVD score (*P* < 0.05), but there were no significant differences between the groups in terms of Fazekas scores. Furthermore, there were no significant difference in the total CSVD scores and Fazekas scores when compared between patients with or without severe symptomatic extracranial arterial stenosis or occlusion.

**Conclusion:**

Severe stenosis or occlusion of the contralateral extracranial artery may increase the incidence of CSVD in patients with LAA cerebral infarction. Active and effective clinical intervention following comprehensive evaluation should be undertaken for unilateral cerebral infarction patients with severe stenosis or occlusion of the contralateral extracranial arterial.

## Introduction

Stroke is one of the leading causes of death and disability around the world (1). Atherosclerotic extracranial artery stenosis or occlusion is a common cause of ischemic stroke. Large artery atherosclerotic (LAA) cerebral infarction was found to be the most common type of stroke by the Trial of ORG 10,172 in Acute Stroke Treatment (TOAST) classification scheme for ischemic stroke etiology (2) and is often accompanied by different degrees of cerebral small vessel disease (CSVD) (3). CSVD accounts for 20–25% of all ischemic strokes (4). Thus, early control or intervention with regards to the occurrence and development of CSVD can have a significant impact on the treatment, prognosis and recurrence of stroke.

The imaging features of CSVD includes lacunae, new subcortical infarcts, white matter lesions, enlargement in perivascular spaces, cerebral microbleeds and brain atrophy (5). Neuroimaging studies have confirmed that CSVD is related to stroke recurrence in patients with LAA cerebral infarctions (6). In particular, a more severe degree of extracranial arterial stenosis (ECAS) has been found to be associated with more severe CSVD in patients with ischemic stroke originating from small or large arteries (3). Moreover, ECAS and CSVD usually share common vascular risk factors including hypertension and age (7, 8); furthermore, both of these factors can influence the outcome of stroke. A previous study showed that ECAS (>50%) doubled the incidence of ipsilateral stroke (9). Wang et al. recently described a clear relationship between ECAS and the new occurrence of cardiovascular and cerebrovascular disease, especially in terms of brain infarction events (10). The association of ECAS with CSVD markers has been explored previously (11, 12); however, the relationship between extracranial vascular stenosis, especially severe extracranial arterial stenosis or occlusion, and the total magnetic resonance imaging ( (MRI) burden of CSVD in patients with ischemic stroke has yet to be addressed.

ECAS is a common problem in the elderly and is one of the most important risk factors for CSVD. Lu et al. recently described ultrasound evidence to suggest that high levels of ECAS were associated with coexisting advanced CSVD in ischemic stroke patients that were suspected of originating in the small or large artery (6). Although patients with differing degrees of ECAS were enrolled to investigate whether high levels of ECAS were related with coexisting CSVD in the study reported by Lu et al. (6), it is unclear as to whether extracranial arterial imaging can help to reduce the burden of CSVD. Therefore, in the present study, we aimed to further investigate the relationship between severe extracranial arterial stenosis or occlusion and CSVD in patients with LAA cerebral infarction.

## Materials and methods

### Study population

This study was a retrospective study. A total of 128 consecutive patients aged between 40 and 80 years with ischemic strokes that were caused by large artery atherosclerosis (according to the TOAST classification scheme) (1) and admitted to our rehabilitation department between January 2021 and July 2022 were recruited within 2–4 weeks of symptom onset. All the patients enrolled provided signed and informed consent.

The exclusion criteria included patients with other types of ischemic stroke according to the TOAST etiology classification scheme and patients with leukoencephalopathy caused by other factors (i.e., multiple sclerosis, toxic encephalopathy and infections). Patients who were unable to complete baseline testing or had contraindications for MRI were also excluded.

### Imaging assessment

All patients received brain MRIs. The MRI scan sequences included T1-weighted imaging (T1WI), T2-weighted imaging (T2WI), diffusion-weighted imaging (DWI), fluid-attenuated inversion recovery (FLAIR) and susceptibility weighted imaging (SWI). All of the included patients were assessed for bilateral ECAS by color Doppler ultrasonography in the bilateral extracranial cerebral arteries, including the common carotid artery, extracranial internal carotid artery (ICA) and the proximal vertebral artery (VA) (ostium, V2–3 segments).

As there are some differences between white matter lesions with regards to total CSVD scores and Fazekas scores only, we further evaluated the total CSVD scores and Fazekas scores in this study.

### Evaluation of total CSVD burden

Total CSVD scores incorporated the lacunes, enlarged perivascular spaces (EPVS), cerebral microbleeds (CMBs) and white matter lesions (WML). CSVD scores ranged from 0–4; the higher the score, the more severe the CSVD (13). The scoring method involved several aspects: (1) Lacunae: one point was awarded on the SVD scale for the presence of 1 or more lacunae; (2) CMBs: one point was awarded on the SVD scale for the presence of 1 or more microbleeds; (3) EPVS: one point was awarded on the SVD scale for moderate to severe (grade 2–4) (14) basal ganglia perivascular spaces, and (4) WML: one point awarded on the SVD scale for confluent deep WMH [Fazekas score 2 or 3 (15)] and/or irregular periventricular WMH extending into the deep white matter (Fazekas score 3).

## The Fazekas scale (0–6)

The Fazekas scale was graded from 0 to 6. Periventricular and deep white matter lesions were scored separately and then combined to generate a total score. Periventricular hyperintensity: 0 points for no lesions; 1 point for cap-like or pencil-like thin-layer lesions; 2 points for lesions with smooth halos; 3 points for irregular periventricular hyperintensities that extended into the deep white matter. Deep white matter signal: 0, no lesions; 1, punctate lesions; 2, lesion fusion; 3, large lesion fusion (15).

According to the North American Symptomatic Carotid Endarterectomy (NASCET) criteria (16), the severity of vascular stenosis was divided into no stenosis, mild stenosis (less than 30%), moderate stenosis (30–69%) and severe stenosis (70%-99%), or occlusion (100%).

### Grouping

**Figure 1:**
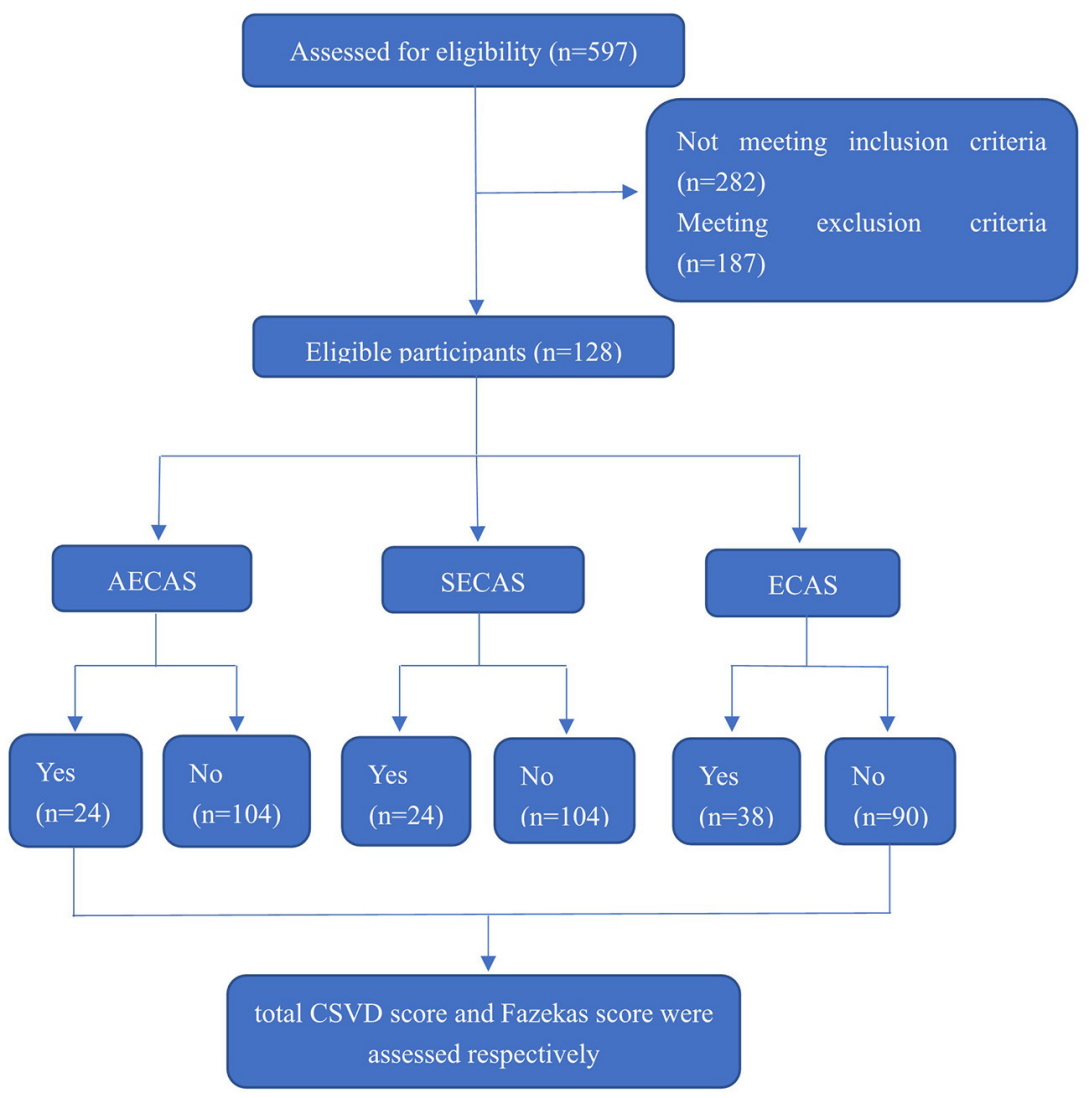
Enrollment and outcomes. CSVD, cerebral small vessel disease; AECAS, asymptomatic extracranial artery stenosis; SECAS, symptomatic extracranial arterial stenosis; ECAS, extracranial artery stenosis.

The design of whole research is showed in Figure 1 and we performed several different comparisons in this study as following:

First, we compared total CSVD scores and Fazekas scores between groups with or without severe extracranial arterial stenosis or occlusion (including severe symptomatic extracranial arterial stenosis and severe asymptomatic extracranial arterial stenosis).

Next, we compared total CSVD scores and Fazekas scores between groups with or without severe symptomatic extracranial arterial stenosis or occlusion.

Finally, we compared total CSVD scores and Fazekas scores in the group with or without severe asymptomatic extracranial arterial stenosis or occlusion.

As an additional note, “symptomatic extracranial arterial stenosis” refers to ipsilateral ECAS in patients with LAA cerebral infarction that directly led to the occurrence of a lesion while “asymptomatic extracranial arterial stenosis” refers to ECAS on the non-lesional side of patients with LAA cerebral infarctions (6).

### Statistical analysis

We compared baseline factors between patients with or without severe symptomatic extracranial arterial stenosis or occlusion, with or without severe asymptomatic extracranial arterial stenosis or occlusion and with or without severe extracranial arterial stenosis or occlusion. These comparisons were performed with Fisher's exact test (for percentages). Statistical analysis and graphing were performed by GraphPad Prism 8.0 (GraphPad Software, Inc., San Diego, California, USA). Continuous variables (such as age) are expressed as the mean ± standard deviation for normally distributed data; independent sample *t-*tests were applied for between-group comparisons. Non-normally distributed data (such as the total CSVD score and the Fazekas score) were expressed as the median and interquartile range or M (IQR) and the Mann Whitney *U* test was performed to evaluate the between-group differences of patients in different groups. A *P* value < 0.05 was considered statistically significant.

## Results

This study included 128 patients with LAA cerebral infarctions, including 92 males and 36 females. Thirty-eight patients had severe extracranial arterial stenosis or occlusion, including 23 patients with severe stenosis or occlusion of the anterior circulation (including the carotid artery and the extracranial segment of the internal carotid artery) and 15 cases of severe stenosis or occlusion of posterior circulation (i.e., within the extracranial segment of the vertebral artery). Among the three groups (with or without severe symptomatic extracranial arterial stenosis or occlusion, with or without severe asymptomatic extracranial arterial stenosis or occlusion, and with or without severe extracranial arterial stenosis or occlusion), there was no significant differences in terms of age, gender, lesion site (anterior circulation or posterior circulation), the history of hypertension, diabetes or stroke (*P* > 0.05) (Table 1).

**Table 1:** Patient comparisons.

**Group**	**Age ($\bar{x}$ ±s)**	**Sex (n)**		**Site (n)**		**Hypertension (n)**		**Diabetes (n)**		**Stroke (n)**	
		**M**	**F**	**A**	**P**	**Yes**	**No**	**Yes**	**No**	**Yes**	**No**
**SECAS**											
Yes	64.50 ± 10.16	18	6	14	10	18	6	10	14	6	18
No	63.60 ± 9.84	74	30	65	39	77	27	48	56	31	73
**AECAS**											
Yes	67.29 ± 9.63	18	6	16	8	18	6	14	10	8	16
No	62.95 ± 9.78	74	30	63	41	77	27	44	60	29	75
**ECAS**											
Yes	65.37 ± 9.06	30	8	23	15	27	11	17	21	9	29
No	63.09 ± 10.15	62	28	56	34	68	22	40	50	28	62

SECAS, symptomatic extracranial arterial stenosis; AECAS, asymptomatic extracranial artery stenosis; ECAS, extracranial artery stenosis; M, male, F, female; A, anterior circulation; P, posterior circulation. *P* > 0.05.

### The total CSVD scores

**Figure 2:**
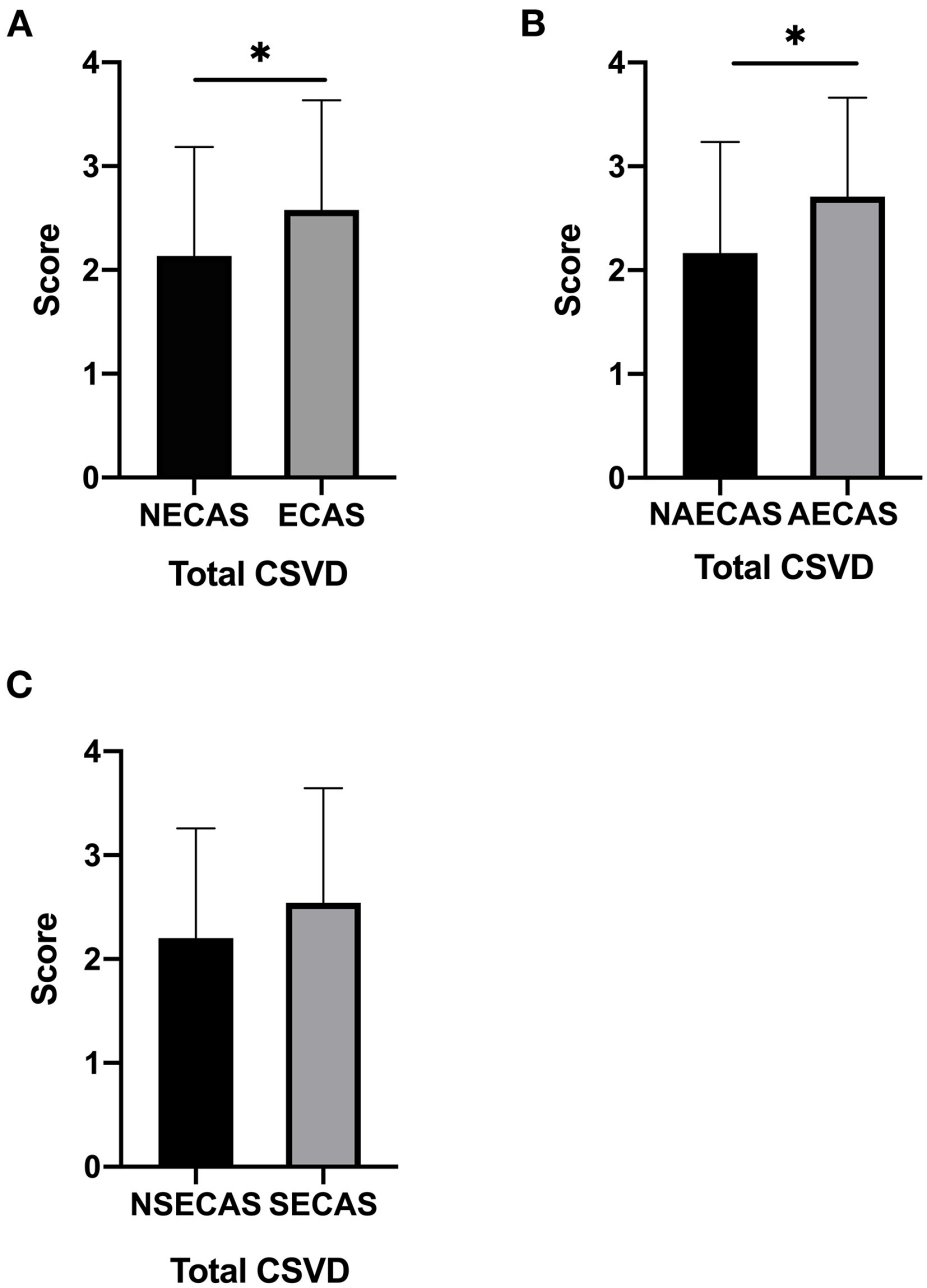
**(A–C)** The total cerebral small vessel disease (CSVD) score in patients with or without severe extracranial arterial stenosis or occlusion, with or without severe symptomatic extracranial arterial stenosis or occlusion, and with or without severe asymptomatic extracranial arterial stenosis or occlusion. ^*^P < 0.05 was considered to indicate statistical significance. SECAS, symptomatic extracranial arterial stenosis; AECAS, asymptomatic extracranial artery stenosis; ECAS, extracranial artery stenosis; NSECAS, no symptomatic extracranial arterial stenosis; NAECAS, no asymptomatic extracranial artery stenosis; NECAS, no extracranial artery stenosis.

The total CSVD scores in patients with severe extracranial arterial stenosis or occlusion significantly higher than those without (*P* = 0.031, Figure 2A); the total CSVD scores in patients with severe asymptomatic extracranial arterial stenosis or occlusion were significantly higher than those without (*P* = 0.024, Figure 2B); but there was no significant difference in total CSVD scores in patients with or without severe symptomatic extracranial arterial stenosis or occlusion (*P* > 0.05, Figure 2C).

### Fazekas scores

**Figure 3:**
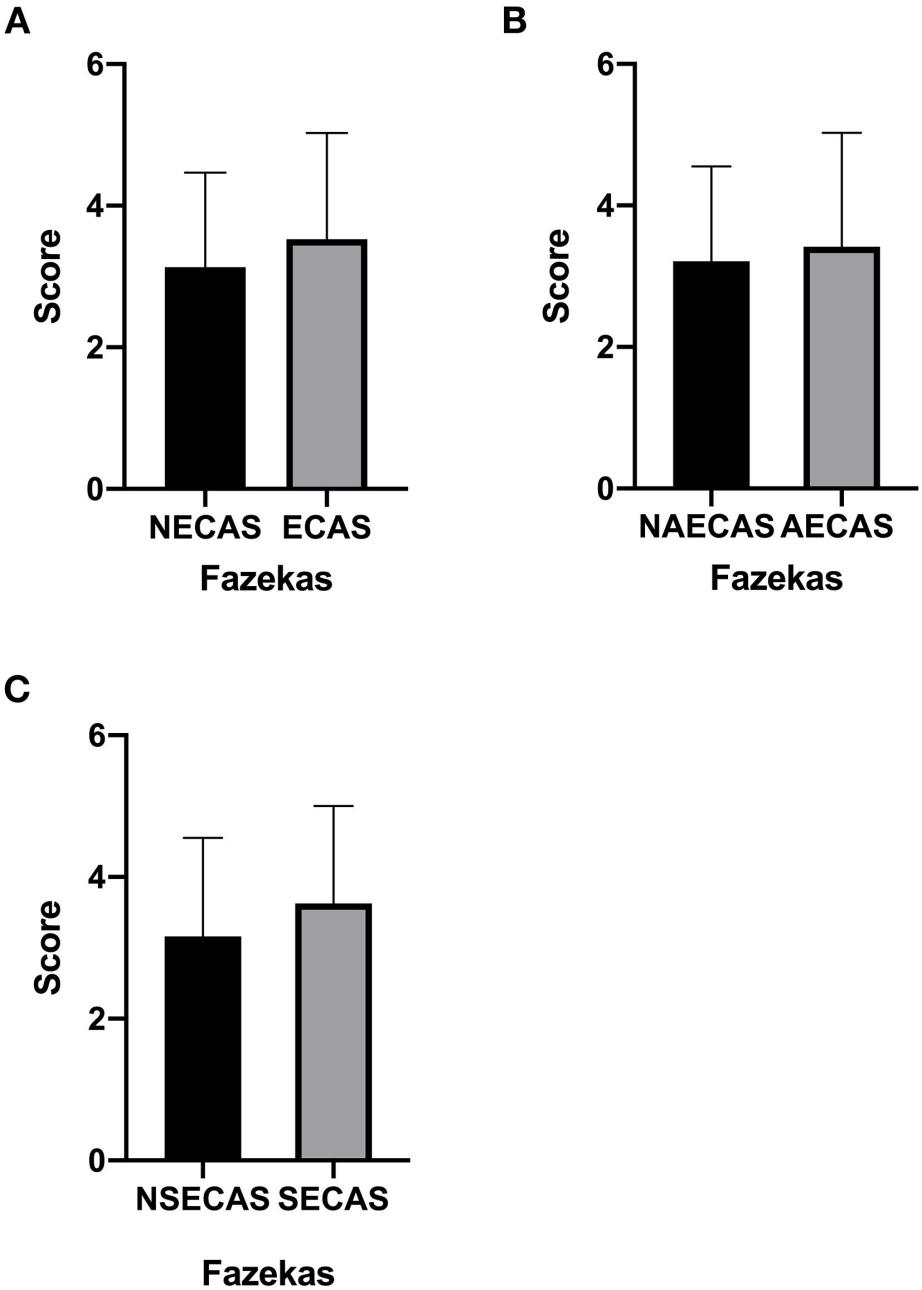
**(A–C)** The Fazekas score in patients with or without severe extracranial arterial stenosis or occlusion, with or without severe symptomatic extracranial arterial stenosis or occlusion, and with or without severe asymptomatic extracranial arterial stenosis or occlusion. *P* < 0.05 was considered to indicate statistical significance. SECAS, symptomatic extracranial arterial stenosis; AECAS, asymptomatic extracranial artery stenosis; ECAS, extracranial artery stenosis; NSECAS, no symptomatic extracranial arterial stenosis; NAECAS, no asymptomatic extracranial artery stenosis; NECAS, no extracranial artery stenosis.

There was no significant difference in Fazekas scores in patients with or without severe extracranial arterial stenosis or occlusion, with or without severe asymptomatic extracranial arterial stenosis or occlusion and with or without severe symptomatic extracranial arterial stenosis or occlusion (*P* > 0.05, Figures 3A–C).

## Discussion

In the current study, we found that patients with severe extracranial arterial stenosis or occlusion, and patients with severe asymptomatic extracranial arterial stenosis or occlusion had significantly higher total CSVD scores. The main results of the present study show that severe stenosis or occlusion of contralateral extracranial artery can promote the occurrence and development of CSVD. Furthermore, our findings further suggest that active and effective clinical intervention following comprehensive evaluation should be undertaken for unilateral cerebral infarction patients with severe stenosis or occlusion of the contralateral extracranial artery.

The imaging hallmarks of CSVD include lacunae, small new subcortical infarcts, white matter lesions, enlarged perivascular spaces, cerebral microbleeds and brain atrophy (4). CSVD has been reported to be preferentially associated with ECAS in patients with ischemic stroke. An article published in 2015 concluded that white matter hyperintensity (WMH) on the ipsilateral side of the internal carotid artery stenosis (ICAS) and the enlarged perivascular space (EPVS) in the basal ganglia region were significantly more severe than in the contralateral hemisphere. Chronic hypoperfusion caused by ICAS is thought to be the predominant mechanism underlying these changes (17). Some studies have also found that the higher the degree of carotid artery stenosis, the more obvious the deep white matter lesions (18). Recent literature shows that the clinical manifestations of CSVD mainly include cognitive decline, abnormal gait, abnormal urinary function, abnormal mental and emotional disorders, and acute stroke manifestations (19). In particular, Staals et al. proposed that total CSVD scores can better assess the overall impact of CSVD on brain function than a single imaging phenotype (13). In addition, total CSVD scores have also been shown to be associated with a poor prognosis in patients with acute cerebral infarction after intravenous thrombolysis (20), and also with a poor long-term neurological prognosis in patients with LAA-type cerebral infarction (21). It appears that ECAS may affect stroke outcomes by affecting CSVD. However, it remains unclear as to whether extracranial arterial imaging can help to reduce CSVD burden. For this purpose, we attempted to explore the relationship between severe extracranial arterial stenosis or occlusion and CSVD in patients with LAA cerebral infarction. Further analysis showed that patients with severe asymptomatic extracranial arterial stenosis or occlusion had significantly higher total CSVD scores than those without. However, there were no significant differences in Fazekas scores. This may be because ECAS is more likely to cause damage to brain function by affecting multiple CSVD imaging phenotypes than single white matter damage. Asymptomatic carotid artery stenosis refers to ECAS in persons without a history of ischemic stroke, transient ischemic attack or other neurological symptoms (22). Asymptomatic extracranial arterial stenosis (AECAS) has previously been used to refer to the same process as asymptomatic carotid artery stenosis; however, in the present study, AECAS refers to the ECAS on the non-lesional side in patients with LAA cerebral infarction. ECAS, and especially AECAS, may promote the occurrence and development of CSVD through chronic hypoperfusion (18) along with the presence of collateral circulation in severe asymptomatic internal carotid artery stenosis (19). CSVD has a significant impact on the treatment, prognosis and recurrence of LAA cerebral infarction. Clinically, active, appropriate, and effective intervention for extracranial artery stenosis may help to reduce or prevent CSVD and thus further reduce the adverse effects of CSVD on brain function. Our study also showed no significant between-group differences in total CSVD scores and Fazekas scores in patients with or without severe symptomatic extracranial arterial stenosis. Based on the previously described mechanisms of ECAS in the promotion of the occurrence and development of CSVD, we believe that, compared with AECAS, symptomatic extracranial arterial stenosis (SECAS) might be given more active and effective interventions during the occurrence of acute stroke events by either reducing hypoperfusion injury or slowing the establishment of collateral circulation and thereby delaying the occurrence and development of CSVD.

However, our study still has some limitations that need to be addressed. First, the manifestations of CSVD are long-term and chronic developmental processes. A cross-sectional or baseline analysis alone may not provide the most accurate description of the relationship between ECAS and the two different processes. Thus, future studies should involve longitudinal follow-up. Second, this study only explored the relationship between CSVD and ECAS without intervention. We do not know the long-term changes of the manifestations of CSVD after appropriate interventions with ECAS. In fact, given that severe stenosis or occlusion of the contralateral extracranial artery may increase the incidence of CSVD, the application of interventions with ECAS may have therapeutic potential on the brain and arterial imaging results of our study.

In conclusion, severe extracranial arterial stenosis or occlusion, and especially severe asymptomatic extracranial arterial stenosis or occlusion, are more likely to promote the occurrence and development of CSVD. These findings differ from other research studies that focused on all degrees of ECAS. Since ECAS and CSVD can both influence the outcome of stroke, providing intervention early within the development of CSVD may have a significant impact on the treatment, prognosis and recurrence of stroke and may also reduce morbidity and mortality. Clinical interventions based on a comprehensive evaluation may need to be carried out for severe extracranial arterial stenosis or occlusion, especially for severe asymptomatic extracranial arterial stenosis or occlusion.

## Data availability statement

The raw data supporting the conclusions of this article will be made available by the authors, without undue reservation.

## Ethics statement

The studies involving human participants were reviewed and approved by the Ethics Committee of Beijing Tiantan Hospital, Capital Medical University. The patients/participants provided their written informed consent to participate in this study.

## Author contributions

PD: conceptualization, formal analysis, data curation, and writing-original draft. H-xY: methodology and formal analysis. Z-xW: methodology and data curation. S-hL: data curation. G-qX: writing-review and editing and funding acquisition. All authors contributed to the article and approved the submitted version.

## Funding

This research was supported by the National Natural Science Foundation of China (Grant Nos.: 82072548 and 82272588).

## Conflict of interest

The authors declare that the research was conducted in the absence of any commercial or financial relationships that could be construed as a potential conflict of interest.

## Publisher's note

All claims expressed in this article are solely those of the authors and do not necessarily represent those of their affiliated organizations, or those of the publisher, the editors and the reviewers. Any product that may be evaluated in this article, or claim that may be made by its manufacturer, is not guaranteed or endorsed by the publisher.
